# Harsh parenting and non-suicidal self-injury in adolescence: the mediating effect of depressive symptoms and the moderating effect of the COMT Val158Met polymorphism

**DOI:** 10.1186/s13034-021-00423-0

**Published:** 2021-11-23

**Authors:** Jinmeng Liu, Xia Liu, Hui Wang, Yemiao Gao

**Affiliations:** Institute of Developmental Psychology, Faculty of Psychology, Beijing Normal University, Beijing, 100875 China

**Keywords:** Non-suicidal self-injury, Harsh parenting, COMT Val158Met polymorphism, Depressive symptoms

## Abstract

**Background:**

Previous studies have suggested that negative parenting environments, especially harsh parenting, are a specific risk factor for non-suicidal self-injury (NSSI). However, the potential mechanism between harsh parenting and NSSI has not been explored. Based on the experiential avoidance model and empirical research, we aimed to examine whether depressive symptoms are a mediator between harsh parenting and NSSI. Moreover, the catechol-*O*-methyltransferase (COMT) Val158Met polymorphism related to depressive symptoms may also exert a moderating effect on NSSI; thus, the interaction between harsh parenting and COMT was also considered in our study.

**Methods:**

A total of 373 junior high school students were recruited for the study by using a longitudinal design. The adolescents answered self-report questionnaires and provided saliva samples for DNA genotyping.

**Results:**

The results revealed that harsh parenting was positively associated with NSSI after 24 months, and this association was mediated by depressive symptoms. Moreover, the moderating role of COMT in the direct and indirect effects of harsh parenting on NSSI was observed only among adolescents with two Val alleles and the relationship was not significant for Met carriers.

**Conclusions:**

Genetic variations of COMT Val158Met may be a critical candidate in understanding the development of depression and NSSI. We conclude that Val homozygotes of the COMT Val158Met polymorphism play a role in susceptibility to both depressive symptoms and NSSI.

## Introduction

Non-suicidal self-injury (NSSI) refers to the deliberate and direct destruction of one’s own body tissue without conscious suicidal intent [1]. It has consistently been reported to be associated with a variety of emotional or borderline personality disorders and an increased risk of suicide [2, 3]. The onset and prevalence of NSSI are especially common in adolescence. A recent meta-analysis showed an NSSI lifetime prevalence of 17.2%, 13.4% and 5.5% in adolescents, young adults, and adults, respectively [4]. NSSI behaviour in Chinese adolescents is as estimated to be as high as 22.4–29% [5, 6]. Clearly, adolescent NSSI is a serious public health concern worldwide [7], and it is important to identify the potential mechanisms and aetiology of NSSI during this period of life.

Harsh parenting refers to a wide range of aversive parenting behaviours, including physical (e.g., spanking, slapping, or hitting) and verbal punishment (e.g., yelling and cursing) at children who have done something wrong [8, 9]. According to Linehan [10], exposure to harsh parenting may influence the likelihood of engaging in NSSI behaviours. Victor et al. [11] also emphasized that harsh parenting, such as shouting, swearing, and spanking, predicted increased odds of subsequent adolescent NSSI onset in a longitudinal design. Although previous research has investigated the linkage between harsh parenting and NSSI in Western countries [12, 13], research into this issue is still rare in China. As a Chinese proverb, “Beating and scolding is the emblem of love”, in the traditional Chinese cultural context, harsh parenting behaviours are generally considered to reflect an indication of parental involvement, concern, and love; therefore, harsh punishment is still adopted by approximately 50% of parents in China [14–16]. Considering the cultural differences, it is necessary to further investigate the relationship between harsh parenting and NSSI in China.

Depressive symptoms have been frequently identified as a mediator of interpersonal risk factors and NSSI. NSSI is often considered to be an emotion-regulation strategy to decrease youth’s emotional distress by distracting from intense emotion through the sight of blood, the sensation of pain, or a focus on the injury itself [1, 17]. The experiential avoidance model proposed that NSSI is maintained by negative reinforcement as a way of escape from unwanted emotional experiences [18]. Consistent with this model, individuals who experience higher levels of anhedonia and depression are more likely to report a history of NSSI [19, 20]. Many empirical studies have revealed a mediating role of depression between interpersonal stressors and NSSI among adolescents. Recent findings from a longitudinal study showed that depressive symptoms play a mediating role in the association between peer bullying and NSSI [21]. Meanwhile, Madjar et al. [22] also found that a sense of loneliness in school could increase the risk of NSSI by increasing the severity of depressive symptoms. Although previous studies have indicated correlations of harsh parenting and depressive symptoms with NSSI [20, 23, 24], no research has explicitly addressed the mechanisms underlying harsh parenting, depression, and NSSI, and especially there is a lack of evidence from longitudinal studies. To bridge these research gaps, we conducted a three-wave longitudinal study to evaluate the longitudinal associations among harsh parenting, depressive symptoms, and NSSI in a community sample of Chinese adolescents.

Brodsky [25] proposed a modified diathesis-stress model for suicide and NSSI by combining findings from the fields of biology, neurology, and genetics, which provides a specific framework to understand how gene–environment (G × E) interactions correlate with suicidal or NSSI behaviours. This model suggests that individuals with “vulnerable genes” tend to develop behavioural problems, especially in stressful environments. Furthermore, the model emphasized the underlying mediating and possible causal mechanisms between gene-environment interactions and NSSI. Specifically, the model suggests that adverse caregiving environments interact with genetic factors to contribute to vulnerability to NSSI, not only through certain character traits (e.g., emotional dysregulation/pessimism) but also through the biological impact on genetic phenotypes and neurotransmitter systems. This means that the interaction between environmental and genetic factors could impact NSSI directly and could also impact the expression of biology or traits such as depressive symptoms, thereby increasing the risk of NSSI.

According to Brodsky [25], not all individuals who experience harsh parenting and depression engage in NSSI, and genes are an important biological underpinning of individual differences in the risk of developing NSSI. Therefore, it is necessary to explore genetic moderators in the relationship between harsh parenting, depressive symptoms and NSSI. However, through Brodsky’s framework, previous studies mostly considered the genetic factor of suicide, and only a few studies have identified the genetic factors related to NSSI, such as the serotonin transporter gene (5-HTTLPR) and brain-derived neurotrophic factor (BDNF Val66Met) gene [26, 27]. Catechol-O-methyltransferase (COMT) is located on chromosome 22q11.1-q11.2 [28] and it plays an important role in regulating an individual’s processing of emotion and cognition by inactivating catecholamine neurotransmitters. The activity of the COMT enzyme is influenced mainly by a functional single nucleotide polymorphism (rs4680; G to A) in the COMT coding region that causes a Val158Met aminoacid substitution in the corresponding protein, with the Val allele exhibiting a 3- to 4-fold increase in enzyme activity compared to the Met allele [29, 30]. Considering that the COMT Val158Met gene is associated with several traits related to NSSI behaviours, including emotion regulation [31, 32], borderline personality disorders [33, 34] and suicidal behaviours [35, 36], we hypothesize that the COMT Val158Met gene might directly moderate the relationship between harsh parenting and NSSI.

In addition, there are numerous genetic association studies implicating the COMT Val158Met polymorphism in major depression disorder [37–39]. In the context of G × E, COMT Val158Met has been found to interact with environmental variations to predict outcomes related to depression symptoms. For example, a longitudinal study has shown that Val-allele children were more likely to develop depression after exposure to a high-risk nurturing environment [40]. Cao et al. [41] also found that adolescents with the Val/Val genotype were more sensitive to depression when exposed to negative peer relationships. Based on Brodsky’s diathesis-stress models, interactions among adverse caregiving environments and the COMT Val158Met polymorphism could also predict the individual’s characteristic traits, such as depressive symptoms. Thus, it is plausible to assume that the COMT Val158Met gene might also moderate the association between harsh parenting and depressive symptoms.

**Fig. 1:**
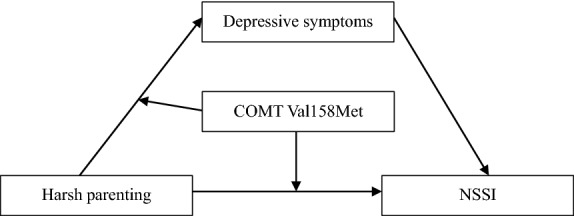
The proposed model of the association between harsh parenting, depressive symptoms, NSSI, and COMT Val158Met

Using a three-wave longitudinal design, the current study aimed to explore the role of depressive symptoms and the COMT Val158Met polymorphism in the relationship between harsh parenting and NSSI. Given that early adolescence has a high rate of NSSI and is a typical onset time of NSSI [1], we investigated a community sample of early adolescents in the current study. Specifically, we constructed a moderated mediation model (see Fig. 1) and proposed the following hypotheses: (1) harsh parenting would be positively associated with NSSI among adolescents; (2) depressive symptoms would mediate the relation between harsh parenting and NSSI; and (3) COMT rs4680 would moderate the link between harsh parenting and NSSI and the link between harsh parenting and depressive symptoms in the mediation model of the relationship between harsh parenting and NSSI.

## Methods

### Participants

Participants were recruited from four junior high schools in Guizhou Province, China. We randomly contacted four public junior high schools, and their principals’ approval of the survey was obtained. Then, at each school, four classes from grade 7 were randomly selected. At baseline (T1), 536 adolescents (M age = 12.80 ± 0.84 years, 52.2% girls) were enrolled, and then the participants completed two follow-up assessments that were undertaken at 6 and 24 months from baseline (T2, n = 516, M age = 13.52 ± 0.84 years, 52.9% girls; T3, n = 373, M age = 14.66 ± 0.87 years, 54.2% girls). No significant differences were found in the variables of interest (i.e., harsh parenting, depressive symptoms and NSSI) or other demographic variables, such as age and gender, between adolescents who participated in all assessments and those who did not.

Written informed consent was obtained from each participant and their parents and school principals at each data collection. Meanwhile, all participants were notified that their participation was completely voluntary and that they had the right to withdraw at any time. After completing each survey, each participant received a gift (a pen and a notebook at T1 and T2, a t-shirt at T3) for their participation. This study was approved by the Research Commission of the authors’ university and the principals of the participating schools.

### Measures

#### Harsh parenting

At T1, harsh parenting was assessed using the Chinese version of the Parent–Child Conflict Tactics Scale (CTSPC, [42, 43]). The questionnaire included 18 items, and adolescents responded to the stem question “How do your parents react when you have done something wrong or they truly do not like?” by rating how often (0 = “never” to 6 = “more than 20 times”) their parents acted according to each item. Psychological aggression was assessed by combining five items such as “my parents called me things like ‘stupid’ or ‘lazy’”, and physical assault was assessed by combining thirteen items such as “spanked on bottom with bare hand”. In the present study, harsh parenting was measured by all items, and the items were averaged, with higher scores indicating higher levels of harsh parenting during the past year. Cronbach’s alpha coefficient for this scale was 0.83 at T1.

#### Depressive symptoms

At T1 and T2, adolescent depressive symptoms were assessed using the Centre for Epidemiological Studies Depression Scale for Children (CES-DC [44]). This scale has been successfully applied to children and adolescents in China [45]. The scale includes 20 items, such as “I do not think I can concentrate on my work.” All responses range from 1 (never) to 4 (always). The items were averaged, with higher scores indicating higher levels of depressive symptoms. In the present study, Cronbach’s alpha coefficient for this scale was 0.84 at T1 and 0.87 at T2.

#### Non-suicidal self-injury

At T1, T2 and T3, Non-suicidal self-injury was measured using a shortened and modified version of the Deliberate Self-Harm Inventory (DSHI), which was developed and validated by Gratz [46]. The scale includes 9 items. In the instructions for use of the scale, we asked the participants to report the frequency at which they intentionally injured themselves without wanting to die, such as “I pluck out my hair deliberately” and “Get yourself electrocuted deliberately without it being life-threatening”. Each of the items was rated on a 4-point scale from 0 (never) to 3 (more than 5 times), reflecting the participants’ frequency of self-injury behaviour over the specified time-periods (e.g., lifetime, since the last assessment). Scores were calculated by averaging all of the responses, with higher scores indicating higher levels of NSSI. In the present study, Cronbach’s alpha coefficient for this scale was 0.83 at T1, 0.82 at T2, and 0.88 at T3.

#### Genotyping

Genomic DNA was extracted from the children’s buccal mucosa on a cotton swab using a Tissue DNA Kit (BioTeke Corporation, Beijing, China) at T3. Single nucleotide polymorphism (SNP) genotyping was performed using the MassARRAY system (Sequenom Inc., San Diego, California, USA) by means of matrix-assisted laser desorption ionization time of flight mass spectrometry (MALDI-TOF). The COMT rs4680 polymorphism was amplified by polymerase chain reaction (PCR) with forward primer (ACGTTGGATGTAGGTGTCAATGGCCTCCAG) and reverse primer (ACGTTGGATGTCATGGGTGACACCAAGGAG).

### Data analysis

First, a preliminary analysis was conducted to compute the means, standard deviations, and correlations among the main variables. Second, the χ^2^ test was used to test whether the distributions of the COMT rs4680 genotype fit the Hardy–Weinberg equilibrium. Third, the mediated moderation model displayed in Fig. 1 was examined. Given that the adolescents’ age and gender would account for the individual differences relating to the main variables [20, 47], we controlled for these demographic variables in our statistical analyses. Therefore, T1 harsh parenting as the independent variable, T3 NSSI as the outcome variable, T2 depressive symptoms as the mediator, and COMT Val158Met as the moderator were entered into the mediated moderation model. Age and gender were entered as covariates. Depressive symptoms and NSSI at T1 were also controlled in all subsequent analyses. For the purpose of minimizing multicollinearity, we standardized all of the predictors.

SPSS software version 19.0 was used to perform the preliminary χ^2^ test, and PROCESS macro software (Model 8) in SPSS [48] was used to test the moderated mediation as displayed in Fig. 1. Specifically, in PROCESS, Model 8 was applied to test the mediated moderation displayed in Fig. 1. According to Model 8 [49], the moderated mediating effect was computed using bias-corrected bootstrapping with 5000 samples; a 95% confidence interval (CI) that does not include zero indicated a significant effect. If the mediated moderation was significant, slope tests were conducted afterwards. According to Aiken et al. [50], two values of harsh parenting, including low (one standard deviation below the mean) and high (one standard deviation above the mean) levels, were defined to acquire detailed information.

## Results

### Descriptive statistics and correlations

The allele distribution of the COMT Val158Met polymorphisms was 202 Val/Val individuals (94 male, 108 female), 148 Val/Met (66 male, 82 female) and 23 Met/Met individuals (12 male, 11 female), representing distributions previously observed in Asian samples. No deviations from Hardy–Weinberg equilibrium were detected for the genotypes (*χ*^2^(2) = 0.15, *p* = 0.93). Based on previous studies [41], we coded the COMT genotypes as a two-level Met carrier model (Val/Val = 1, Met/Val, Met/Met = 0).

Table 1 depicts the descriptive statistics and correlations for all study variables. There were no significant associations between the COMT genotypes and harsh parenting, indicating the absence of a correlation between genes and the environment. In addition, the results of the bivariate correlations showed that harsh parenting was positively correlated with NSSI at Time 1 and Time 3, with depressive symptoms at Time 1 and Time 2, respectively.

**Table 1:** Means, standard deviations, and correlations among all variables

Variable	*M*	*SD*	1	2	3	4	5	6	7	8
1. Age (T1)	12.81	0.87	1							
2. Gender^a^	0.44	0.50	0.12*	1						
3. COMT	0.46	0.50	0.01	− 0.01	1					
4. HP (T1)	0.55	0.60	0.06	− 0.004	0.03	1				
5. DS (T1)	1.99	0.47	0.06	− 0.12*	− 0.03	0.39**	1			
6. NSSI (T1)	0.12	0.26	− 0.07	0.03	− 0.02	0.31**	0.30**	1		
7.DS (T2)	2.14	0.50	0.01	− 0.19*	0.05	0.30**	0.58**	0.28**	1	
8. NSSI (T3)	0.25	0.36	− 0.05	− 0.01	0.02	0.17**	0.23**	0.39**	0.34**	1

T1: Time 1; T2: Time 2; T3: Time 3

*HP* harsh parenting, *DS* depressive symptoms, and *NSSI* non-suicidal self-injury

^a^Female = 0, Male = 1

*p < 0.05, **p < 0.01

### Mediated moderation analysis

**Fig. 2:**
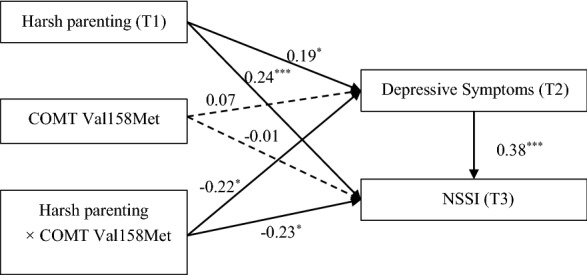
The moderated mediation model among adolescents T1 to T3. The dotted line is not significant; *p < 0.05, **p < 0.01, ***p < 0.001

Table 2 shows a significant longitudinally moderated mediation model for harsh parenting and NSSI relationships. Specifically, as shown in Fig. 2, harsh parenting at T1 was positively associated with NSSI at T3 (*β* = 0.24, *p* < 0.001) and could also significantly positively affect NSSI at T3 through depressive symptoms at T2 (*β* = 0.19, *p* < 0.05; *β* = 0.38, *p* < 0.001), whereas COMT was not associated with depressive symptoms at T2 (*β* = 0.07, *p* > 0.05) or NSSI at T3 (*β* = − 0.01, *p* > 0.05). Furthermore, COMT significantly moderated the impacts of harsh parenting at T1 on NSSI at T3 (*β* = − 0.23, *p* < 0.05) and depressive symptoms at T2 (*β* = − 0.22, *p* < 0.05). The above results showed that the interaction between harsh parenting and COMT directly predicted adolescent future NSSI levels and partially indirectly affected NSSI through depressive symptoms as a mediating variable.

**Table 2:** Testing the moderated mediation model in adolescents

Variable	Depressive symptoms (T2)			NSSI (T3)		
	β	SE	95% CI	β	SE	95% CI
Harsh parenting (T1)	0.19*	0.08	[0.03, 0.35]	0.24***	0.09	[0.06, 0.42]
COMT	0.07	0.10	[− 0.12, 0.26]	− 0.01	0.11	[− 0.23, 0.20]
Harsh parenting (T1) × COMT	− 0.22*	0.10	[− 0.42, − 0.01]	− 0.23*	0.12	[− 0.46, − 0.004]
Depressive symptoms (T2)				0.38***	0.07	[0.24, 0.51]
Age	− 0.02	0.06	[− 0.13, 0.09]	− 0.10	0.06	[− 0.23, 0.02]
Gender	0.29*	0.10	[0.09, 0.48]	− 0.19	0.11	[− 0.41, 0.04]
Depressive symptoms (T1)	0.50***	0.06	[0.39, 0.61]	0.03	0.07	[− 0.11, 0.17]
NSSI (T1)	− 0.10*	0.05	[− 0.20, − 0.004]	0.01	0.05	[− 0.10,0.11]
*R*^2^	0.32			0.19		
*F*	18.30***			7.90***		

*p < 0.05, **p < 0.01, **p < 0.001

**Fig. 3:**
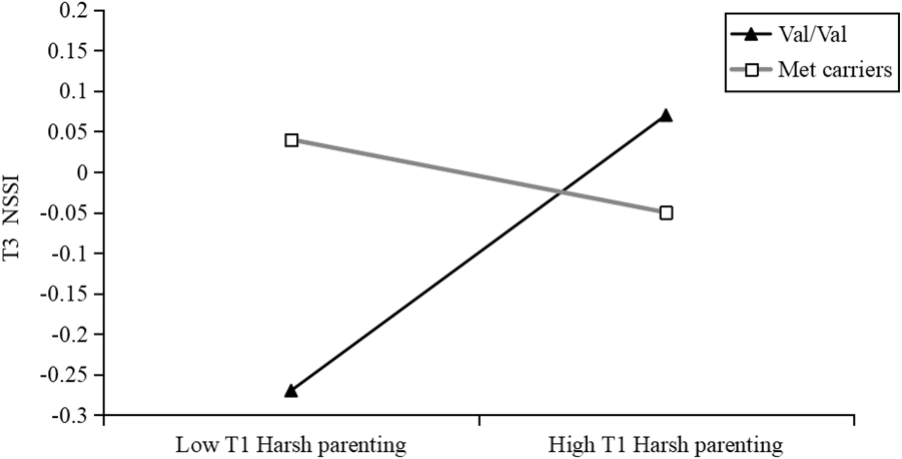
Interactive effect of the COMT genotype and T1 harsh parenting on T3 NSSI

**Fig. 4:**
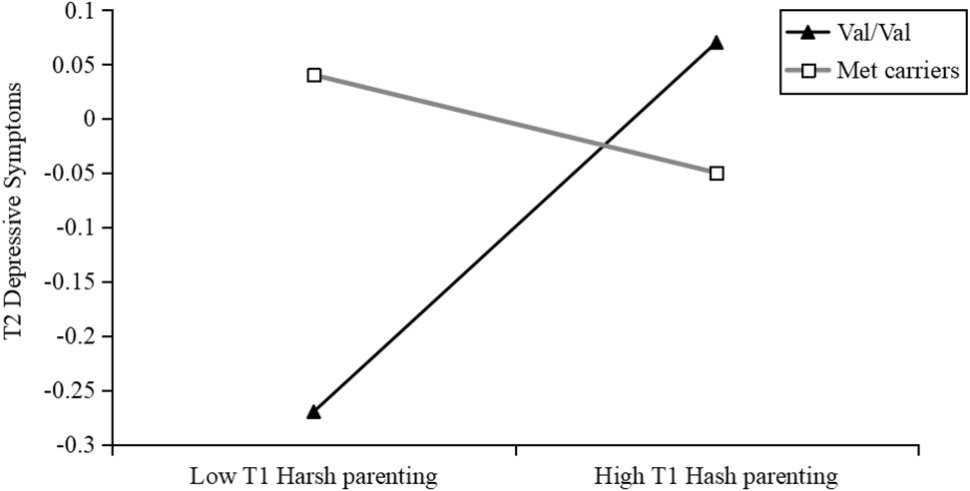
Interactive effect of the COMT genotype and T1 harsh parenting on T2 depressive symptoms

To facilitate the description, a simple slope analysis was conducted to identify the interaction effect between harsh parenting at T1 and COMT on NSSI at T3. As shown in Fig. 3, harsh parenting at T1 positively predicted NSSI at T3 only among individuals with Val/Val genotypes (*b* = 0.30, *t* = 3.23, *p* = 0.001, 95% CI 0.12 to 0.48) compared to Met carriers (*b* = -0.04, *t* = − 0.68, *p* = 0.57, 95% CI − 0.19 to 0.11). Meanwhile, to examine the interaction effect between harsh parenting at T1 and COMT on depressive symptoms at T2, we used the same procedures employed for characterizing the interaction for the prediction of NSSI at T3. As shown in Fig. 4, harsh parenting at T1 positively predicted depressive symptoms only among individuals with Val/Val genotypes (*b* = 0.18, *t* = 2.22, *p* = 0.03, 95% CI 0.02 to 0.34) compared to Met carriers (*b* = -0.05, *t* = − 0.68, *p* = 0.50, 95% CI − 0.18 to 0.09). Overall, the results indicated that the moderating effect of COMT was only significant for Val/Val individuals but not for Met carriers.

## Discussion

Guided by the theoretical model from Brodsky [25], the goal of the current study was to understand how the COMT Val158Met polymorphism interacts with harsh parenting to predict NSSI among adolescents. Using a three-wave longitudinal sample of Chinese adolescents, the results indicated that adolescents exposed to high levels of harsh parenting were associated with more NSSI 24 months later, and depression symptoms partially mediated the association between harsh parenting and NSSI. Moreover, our study found that the COMT Val158Met polymorphism moderates the link between harsh parenting and NSSI and the link between harsh parenting and depression in the mediation model of the relationship between harsh parenting and NSSI. Notably, the current study expands on previous work to emphasize the NSSI aetiology from the genetic and environmental perspectives.

In line with Linehan [10], our findings have identified harsh parenting as a significant interpersonal risk factor for NSSI in Chinese adolescence. This suggests that when designing intervention programs to reduce the incidence of NSSI, special attention should be given to adolescents who experience a longer period of physical or verbal punishment from their parents. More importantly, consistent with the experiential avoidance model [18], we confirmed that depressive symptoms play a mediator role in the association between harsh parenting and NSSI. According to the experiential avoidance model [18], harsh parenting acts as a negative stimulus that elicits unwanted emotional experiences (e.g., sadness, frustration), and NSSI may help escape or avoid depressed affects being experienced as a result of harsh parenting. To our knowledge, this is one of the first studies to investigate this longitudinal mediation mechanism underlying the relationship between harsh parenting and NSSI.

In regard to considering the moderating role of genetics in the associations among harsh parenting, depressive symptoms, and NSSI, our findings contribute to extant research into the negative parenting-NSSI linkage. We found two potential pathways by which COMT plays a moderating role in the relationship between harsh parenting and NSSI. One of the paths was consistent with our hypothesis that COMT moderated the relation between harsh parenting and NSSI directly. Specifically, in very harsh parenting environments, individuals with two Val alleles reported more NSSI 24 months later than Met carriers. Previous studies have demonstrated that the COMT Val/Val genotype is associated with more persistent dopamine degradation and less synaptic dopamine, which might increase flexibility but decrease the stability of neural network activation states, hence rendering adolescents susceptible to negative environments [51]. For example, Kwon et al. [52] indicated that compared to the Met carrier genotype, individuals with the Val/Val genotype showed higher suicidal ideation when they were exposed to a negative environment, such as child abuse. Given that NSSI and suicidal ideation are largely driven by overlapping genetic factors [53], it is possible that the COMT Val158Met polymorphism may be one of the genetic factors to interpret both NSSI and suicidal ideation.

The other path was that COMT moderated the relation between harsh parenting and depressive symptoms. Similar to the direct path, individuals with the Val/Val genotype exhibited more depressive symptoms when exposed to high levels of harsh parenting and fewer depressive symptoms when experiencing less harsh parenting. This result is consistent with prior findings that individuals with two Val alleles may be more susceptible to negative parenting environmental influences and thus develop depression [38, 40]. Although the definitive mechanisms of the Val/Val genotype in being more sensitive to the environment remain an open question, combined with recent empirical studies, we speculate that neurobiological mechanisms may underlie these two interactive effects. Recently, a longitudinal twin study found that exposure to negative parenting would trigger adolescents’ depressive symptoms by increasing the connectivity of the amygdala with the ventrolateral prefrontal cortex, and this neurobehavioural association is heritable during adolescence [54]. Furthermore, a highly expressed COMT genotype (Val) is associated with increased amygdala activity [55, 56]. Thus, negative parenting and COMT may increase the vulnerability to depressive symptoms through their synergistic effects on amygdala circuitry. Taken together, the significant interaction between harsh parenting and the COMT Val158Met polymorphism on both NSSI and depressive symptoms provides some empirical evidence for Brodsky’s diathesis-stress model of suicide and NSSI [25].

Several limitations need to be considered in our study. First, the current study used adolescent self-reports to collect the data, which could be subjected to bias. Therefore, in the future, studies should attempt to also collect data from the children’s parents or other caregivers. Second, although our study is the first to suggest the moderating role of the COMT Val158Met polymorphism in NSSI, the effect of only a single genetic variant on NSSI was examined in this study. Future work should expand the focus to other candidate genes or polygenic risk scores. Third, some studies have found that Asian, Caucasian and Mexican samples differ in their COMT Val158Met polymorphism distribution [57]; thus, the results reported herein should be interpreted carefully in terms of generalization to the overall adolescent population because of the ethnicity and region of our sample (i.e., Chinese Han).

Despite these limitations, the current study has some implications for educational and clinical practitioners. First, harsh parenting is always a familial risk factor for NSSI. Although harsh parenting is still acceptable among Chinese parents, it contributes to later increases in NSSI. To decrease its use, some prevention intervention methods can be used, such as changing parents’ favourable attitudes towards harsh parenting, teaching parents emotion coaching skills, and planning some positive parenting programs [58–60]. Second, it was also found that harsh parenting could increase adolescents’ NSSI by increasing their depressive symptoms, which provides insights into the future development of interventions for NSSI. Specifically, interventions that aim to decrease the depression level caused by harsh parenting may help reduce children’s NSSI. Emotion regulation has been identified as an effective intervention strategy for depression and NSSI [61]. Third, when working with adolescents with histories of maltreatment who also have depression, clinicians should take care to screen for self-injury thoughts and behaviours. Fourth, individual differences in the susceptibility to harsh parenting environments are at least partially genetically influenced during adolescence. Thus, recognizing susceptible individuals is necessary to inform clinical practice and intervention. Last, similar to our findings, other studies also found a negative role of harsh parenting and depressive symptoms and a moderating role of the COMT Val158Met polymorphism in suicide [52, 62]. It is possible that both NSSI and suicide occur in a common continuum [63] and may share similar biological and environmental underpinnings, which suggests that future interventions of NSSI can consider combining them with suicide prevention programs.

## Data Availability

The datasets analysed in the current study are available from the corresponding author on reasonable request.

## Abbreviations

NSSI: Non-Suicidal Self-Injury
COMT: Catechol-O-methyltransferase
